# Draft Genome Sequence of the Most Traditional ε-Poly-l-Lysine Producer, Streptomyces albulus NBRC14147

**DOI:** 10.1128/MRA.01515-18

**Published:** 2019-01-24

**Authors:** Kazuya Yamanaka, Yoshimitsu Hamano

**Affiliations:** a Department of Life Science & Technology, Kansai University, Suita, Osaka, Japan; b Department of Bioscience, Fukui Prefectural University, Yoshida-gun, Fukui, Japan; University of Rochester School of Medicine and Dentistry

## Abstract

The genus Streptomyces is known for its secondary metabolite biosynthetic capacities. We report here the draft genome sequence of the most extensively studied ε-poly-l-lysine producer, Streptomyces albulus NBRC14147. Bioinformatic analysis of the 9.6-Mb chromosome identified a large number of secondary metabolite biosynthetic gene clusters.

## Abstract (precis)

The genus Streptomyces is known for its secondary metabolite biosynthetic capacities. We report here the draft genome sequence of the most extensively studied ε-poly-l-lysine producer, Streptomyces albulus NBRC14147.

## ANNOUNCEMENT

In 1977, Shima and Sakai isolated Streptomyces albulus NBRC14147 (formerly strain 346) from soil as a producer of ε-poly-l-lysine (ε-PL), which consists of 25 to 35 l-lysine residues, with an isopeptide linkage between its ε-amino and α-carboxyl groups (1). Because this characteristic polymer shows strong antimicrobial activity (2), and its safety has already been demonstrated in experiments using rats (3), it has been widely used as a natural food preservative in a number of countries, including Japan, the United States, South Korea, and China. To date, a number of ε-PL-producing actinomycetes have similarly been isolated from soil. Since the most traditional producer, S. albulus NBRC14147, has the longest history, most of the trailblazing studies on the microbial production and biosynthesis of ε-PL focused on this particular strain or its derivatives (4–6). However, its genomic information, which allows for rational metabolic engineering, has yet to be revealed. In addition, its potential to produce secondary metabolites other than homo poly-amino acids has remained unclear. We thus interrogated the genome of the most extensively studied ε-PL producer, S. albulus NBRC14147.

The genomic DNA of S. albulus NBRC14147 was extracted from cultures grown for 2 days at 30°C in sucrose contained Luria-Bertani (SLB) medium (7) using standard procedures (8). The extracted genome was further purified using a PowerClean DNA Clean-Up kit (Mo Bio) and then sheared into 10-kb fragments by using a g-Tube (Covaris). A genomic library generated with the SMRTbell adaptor was fluorophotometrically assayed using the Quant-iT double-stranded DNA (dsDNA) broad-range (BR) assay kit (Invitrogen) and was then analyzed using PacBio single-molecule real-time (SMRT) sequencing technology (9). Four SMRT cells employing a P4 polymerase and C2 chemistry combination (P4-C2) run on a Pacific Biosciences RS II instrument produced 396,562 reads totaling 1.2 Gb, which corresponds to an approximately 129-fold coverage of the genome. A *de novo* assembly was performed using RS Hierarchical Genome Assembly Process (HGAP) in SMRT Analysis software version 2.1.1, based on PreAssembler version 1 (6,000-bp cutoff for seed reads), Celera assembler version 1, and Quiver polishing scripts with the “only unambiguously mapped reads” option, yielding 8 contigs with an *N*_50_ value of 5,440,599-bp. The genome of S. albulus NBRC14147 is 9.6 Mb in size, with a G+C content of 72.2%; these values are highly similar to those of the other S. albulus strains previously sequenced (10–12).

Annotation using the DFAST Legacy server (13), based on Prokka (14), with a RefSeq database allowed the identification of 98 tRNA genes and 8,419 predicted protein coding genes, including genes organizing the canonical lysine biosynthetic diaminopimelate pathway, which could be involved in ε-PL biosynthesis. Analysis of the chromosome using antiSMASH 4.0 (15) predicted 37 biosynthetic gene clusters (BGCs) for secondary metabolite production, implying that the strain could possess considerable secondary metabolite biosynthetic potential. The NBRC14147 chromosome appears to harbor several BGCs for uncharacterized nonribosomal peptides and polyketides. The draft genome sequence will allow us not only to genetically and metabolically engineer this valuable strain to further improve ε-PL production but also to explore uncharacterized natural product drug leads.

### Data availability.

This whole-genome shotgun project has been deposited in DDBJ/EMBL/GenBank under the accession no. BHXC00000000. The version described in this paper is the first version, BHXC01000000. The raw sequence reads have been deposited in DDBJ/EMBL/GenBank under the accession no. DRR161053.
